# Microplastics Determination in Gastrointestinal Tracts of European Sea Bass (*Dicentrarchus labrax*) and Gilt-Head Sea Bream (*Sparus aurata*) from Tenerife (Canary Islands, Spain)

**DOI:** 10.3390/polym14101931

**Published:** 2022-05-10

**Authors:** Raquel Sánchez-Almeida, Cintia Hernández-Sánchez, Cristina Villanova-Solano, Francisco Javier Díaz-Peña, Sabrina Clemente, Javier González-Sálamo, Miguel González-Pleiter, Javier Hernández-Borges

**Affiliations:** 1 Departamento de Química, Unidad Departamental de Química Analítica, Facultad de Ciencias, Universidad de La Laguna (ULL), Avda. Astrofísico Fco. Sánchez, s/n, 38206 San Cristóbal de La Laguna, Spain; rachelschez@gmail.com (R.S.-A.); cvillano@ull.edu.es (C.V.-S.); jgsalamo@ull.edu.es (J.G.-S.); 2 Departamento de Obstetricia y Ginecología, Pediatría, Medicina Preventiva y Salud Pública, Toxicología, Medicina Forense y Legal y Parasitología, Área de Medicina Preventiva y Salud Pública, Escuela Politécnica Superior de Ingeniería, Sección de Náutica, Máquinas y Radioelectrónica Naval, Universidad de La Laguna (ULL), Vía Auxiliar Paso Alto, 2, 38001 Santa Cruz de Tenerife, Spain; 3 Instituto Universitario de Enfermedades Tropicales y Salud Pública de Canarias, Universidad de La Laguna (ULL), Avda. Astrofísico Fco. Sánchez, s/n, 38206 San Cristóbal de La Laguna, Spain; 4 Departamento de Biología Animal, Edafología y Geología, Avda. Astrofísico Fco. Sánchez, s/n, 38206 San Cristóbal de La Laguna, Spain; fjdiazpe@ull.edu.es (F.J.D.-P.); msclemen@ull.edu.es (S.C.); 5 Department of Chemistry, Sapienza University, P.le Aldo Moro, 5, 00185 Rome, Italy; 6 Department of Biology, Faculty of Sciences, Universidad Autónoma de Madrid, Cantoblanco, 28049 Madrid, Spain; mig.gonzalez@uam.es

**Keywords:** aquaculture, microplastics, *Dicentrarchus labrax*, *Sparus aurata*, Canary Islands, fishing, fourier-transform infrared spectroscopy

## Abstract

Microplastic pollution has an extremely widespread distribution, to the extent that microplastics could be ingested by aquatic organisms, including species of commercial importance for fisheries and aquaculture. In this work, the anthropogenic particles content of the gastrointestinal tracts of 86 individuals of cultivated European sea bass (*Dicentrarchus labrax*, *n* = 45) and gilt-head sea bream (*Sparus aurata*, *n* = 41) from Tenerife (Canary Islands, Spain) was determined. Samples were bought at local markets and directly transported to the laboratory. After the dissection of the fishes and digestion of the gastrointestinal tracts in 10% KOH (*w*/*v*) at 60 °C for 24 h, the digests were filtered (50 µm stainless-steel mesh) and visualized under a stereomicroscope, finding that most of the items were colourless (47.7% for *Dicentrarchus labrax* and 60.9% for *Sparus aurata*) and blue (35.3% vs. 24.8%) microfibers, with an average length of 1957 ± 1699 µm and 1988 ± 1853 µm, respectively. Moreover, 15.3% of the microfibres were analysed by Fourier transform infrared spectroscopy, showing the prevalence of cellulosic fibres together with polyester, polyacrylonitrile, and poly(ether-urethane). This pattern (microplastics shapes, colours, sizes, and composition) clearly agrees with previous studies carried out in the Canary Islands region regarding the determination of microplastics in the marine environment.

## 1. Introduction

Microplastic (MP) pollution is undoubtedly one of the most important environmental problems that humans have to face, impacting the marine environment, the air, and soils, and also increasingly found in the biota [1,2]. In the marine environment, where most studies on MPs contamination have focused since the first reports were published in the 1970s [3,4,5], special attention is paid to the presence of MPs in living organisms such as fish, which represent an important base of the human diet and show contrasting feeding habits among species. In this sense, MPs ingested by wild or aquaculture fish are closely related to the presence, distribution, and fate of MPs in the environments and, as a result, their monitoring helps to understand potential sources and to take actions to mitigate the problem. This is particularly important in aquaculture systems, in which location, structures, diet, etc. can be controlled. However, up to now, few studies have focused on the monitoring of MPs accumulation in cultivated fish, so it remains necessary to provide more data regarding their occurrence [6,7,8,9,10].

European sea bass (*Dicentrarchus labrax*) is a fish with an elongated and laterally compressed body of silver colour, with large scales. The species can grow to a maximum length of 103 cm, but usually ranges 23–46 cm [11]. It is a marine or brackish demersal species that is distributed in the eastern Atlantic, from Iceland and Norway to Senegal, including the easternmost islands of the Canary Islands archipelago [12], the Mediterranean, and the Black Sea. It inhabits coastal waters up to 100 m deep, but it is more common in shallow waters, occurring in various types of bottoms, often entering estuaries, lagoons, and occasionally in river mouths. In its northern range of distribution, the species enters coastal waters and river mouths in summer but migrates offshore and occurs in deeper waters during winter. Juveniles form small groups and feed on invertebrates, mainly crustaceans and molluscs, while adults appear to be less gregarious and piscivorous [11]. The sea bass has been designated as a very opportunistic species that takes advantage and feed preferentially on the more abundant prey species [13].

Gilt-head sea bream (*Sparus aurata*) is a demersal fish with a laterally compressed body of blue-grey coloration in dorsal view and silver-yellow on its sides. The species is distributed in the eastern Atlantic, from the British Isles, Strait of Gibraltar to Cape Verde and around the Canary Islands, and it also occurs in the Mediterranean and in the Black Sea [11]. It is found in a variety of bottoms, including seagrass beds and sandy bottoms, as well as the surf zone. Specimens are common up to depths of about 30 m, but adults may occur at 150 m depth. This sedentary fish occurs either solitary or in small aggregations, and is mainly carnivorous and accessorily herbivorous, feeding mostly on shellfish, including mussels and oysters [11].

Both European sea bass and gilt-head sea bream are species of great commercial interest. In fact, they are the most important and widely cultivated commercial fishes in the Mediterranean, whose main producers are Greece, Turkey, Spain, Egypt, and Italy [14]. In the particular case of Spain, it is the EU Member State with the largest aquaculture crop, with the most produced species being mussel, European sea bass, gilt-head sea bream, and rainbow trout, a ranking in which the Canary Islands is the second community with the highest production of European sea bass and gilt-head sea bream, only behind the Valencian Community, accounting for 26.5% of Spanish production [14].

To the best of our knowledge, the presence of MPs in specimens of both types of farmed fish has only been reported on very few occasions. This is the case for gilt-head sea bream from Mar Menor Lagoon (Spain) [15] as well as for European sea bass from Spain [7,16] and Greece [17]. Therefore, this work aims to study the presence of MPs in the gastrointestinal tracts of cultivated European sea bass and gilt-head sea bream in Tenerife (Canary Islands, Spain), the two most produced and consumed farmed fish in the region. For this purpose, after digestion of the tracts at 60 °C for 24 h with a KOH 10% (*w/v*) solution, MPs were classified according to their shapes, size, colour, and composition. This study constitutes the second in the literature regarding the assessment of MP ingestion by cultivated fish in the Canary Islands archipelago, and the third in the region concerning fish of any type. The results may be of interest to the fish farming industry with the aim of reducing MPs pollution, while providing information on the potential entry of this contaminant into the food chain through the intake of farmed fish.

## 2. Materials and Methods

### 2.1. Materials and Contamination Control

All material used during the process was plastic-free. To avoid MP contamination of the samples in the laboratory, non-volumetric glassware was covered with aluminium foil and heated up to 550 °C for 4 h in a Carbolite CWF 11/13 muffle (Sheffield, UK), while volumetric glassware was cleaned using NoChromix solution (Godax Laboratories, Cabin John, MD, USA) in sulfuric acid (95% *w*/*w*, VWR International, Radnor, PA, USA) for 24 h. When necessary, fishes and laboratory materials were washed at least three times with Milli-Q water obtained from a Milli-Q A10 gradient system from Millipore (Burlington, MA, USA) and previously filtered through a polyvinylidene fluoride (PVDF) 0.22 μm filter. Milli-Q water was also used to prepare the KOH 10% (*w*/*v*) solution which was filtered through 0.22 μm filters of PVDF.

Special care was taken in the laboratory to minimize airborne microplastic contamination. Sample manipulation (fish dissections, filtration of the digests, etc.) was carried out in a glove box. The air of the laboratory was filtered with an air purifier (Mi Air Purifier 2H, Model:AC-M9-AA, Beijing Smartmi Electronic Technology Co., Ltd, Beijing, China) equipped with a HEPA (High Efficiency Particulate Air) filter (removal efficiency of 99.97% of the particles of ≥0.3 μm size). Orange laboratory coats were used during sample processing and observation to facilitate the identification of possible contamination of the samples caused by the clothing of the operators. Stereomicroscope observation was performed using closed Petri dishes and under a laminar closed flow hood to reduce airborne contamination.

Laboratory controls (full sample pre-treatment without gastrointestinal tracts) were also developed with every batch of samples in order to control laboratory contamination. Additionally, checks for contamination during processing were conducted by exposing filters to the air in the laboratory.

### 2.2. Sample Processing and Observation

This study includes the determination of MPs in the gastrointestinal tracts of two different species of fish which were cultivated in fish farms located at the municipality of Arona, at the southwest of Tenerife, Canary Islands (Spain). A total of 86 specimens, 45 cultured European sea bass and 41 cultured gilt-head sea breams, were bought in local markets of Tenerife from January 2019 to March 2019. Once at the laboratory, fork and standard lengths were measured using a calliper (±0.1 cm) as well as the weight of individuals (±0.1 g). Fishes were then washed with filtered Milli-Q water to remove any possible external contamination. Each specimen was dissected inside a glove box on a metal tray using stainless-steel scissors. The dissection began with a shallow ventral cut from the anus to the in between pelvic fins, to avoid damage to the internal organs, the cut was continued all the way up to the gills. Subsequently, the body wall was moved away to allow the gastrointestinal tracts to be extracted from the upper part of the oesophagus to the intestine. The tracts were weighed on a 0.01 g precision balance with a maximum capacity of 4.2 kg (LP4202-C Model from VWR International) and placed in glass beakers. Batches of 5 samples were simultaneously digested with KOH 10% (*w/v*) for 24 h at 60 °C (INCU-Line Microbiological Incubator/Stove from VWR International). A constant volume of 15 mL of KOH 10% (*w/v*) was used per each 10 g of sample; this ratio was maintained for each sample analysis. Afterwards, the digests were filtered using a vacuum filtration system with a 50 µm stainless steel mesh (AISI-304 mesh filter Labopolis, Alcalá de Henares, Spain) previously washed with filtered Milli-Q water. Once filtered, the filtrates were placed in small glass Petri dishes (55 mm × 14 mm diameter) that were sealed for further studies. The particles were directly visualized on the Petri dishes under a trinocular light stereomicroscope with magnifications ×0.65–×5.5 (Euromex Nexius Zoom EVO, Arnhem, The Netherlands) and equipped with an image analysis system (Levenhuk M1400 PLUS-14 Mpx digital camera with the Levenhuk Lite software version x64, 4.10.17659.20200906) to classify and identify the plastic particles found according to their shapes, colours, and sizes, with the lower limit length of the particles being 50 μm. MPs were classified according to their shapes in fragments, fibres/lines, pellets, microbeads, foams and films. Each particle was photographed, and their sizes were measured. To determine if a particle is made of plastic, the criteria of Hidalgo-Ruz et al. were met [18,19].

### 2.3. MicroFourier-Transform Infrared Spectroscopy Analysis

A randomly distributed subsample of microparticles (*n* = 69, 15.3% of the total found) that included fibres of each filter was analysed by Fourier-transform infrared (µFTIR) spectroscopy using a Perkin-Elmer Spotlight™ 200 Spectrum Two instrument with a mercury cadmium telluride detector. Microparticles were placed on KBr, which was used as a slide, and their spectra were recorded in micro-transmission mode using the following parameters: spot 50 μm, 32 scans, and spectral range 550–4000 cm^−1^. All spectra were compared with Omnic 9.1.26 database (ThermoFisher Scientific Inc., Boston, MA, USA) and with spectra from our database. Microparticles were considered plastics when the match confidence was at least 70%. Natural (cotton and linen) and semi-synthetic fibres (rayon/viscose/cellophane, lyocell/Tencel) as well as cotton and linen with non-natural colours that consist of cellulose, were classified as cellulosic since their spectra are practically identical and, therefore, they are difficult to differentiate, especially in the case of the microparticles found in the environment due to weathering processes. Polyethylene terephthalate (PET) was classified as “polyester” since it is a thermoplastic polymer resin of the polyester.

### 2.4. Statistical Analysis

Statistical methods were implemented using IBM SPSS Statistics V26.0. The level of significance for all tests was set to *p* ≤ 0.05. To detect differences in particles abundances and lengths among fish species, a *t*-test for independent samples was used. The Mann–Whitney non-parametric U-test was applied when parameters did not conform to a normal distribution (Kolmogorov–Smirnov test) and homogeneity of variance (Levene test).

## 3. Results and Discussion

### 3.1. Sampling and Sample Treatment

Table 1 summarizes the fork and standard lengths of the analysed specimens as well as their weight, including that of the gastrointestinal tracts. As can be seen, the average weight of the specimens was 660.5 ± 46.3 g for *Dicentrarchus labrax* and 609.7 ± 125.8 g for *Sparus aurata,* and of the gastrointestinal tracts 11.0 ± 1.6 g for *Dicentrarchus labrax* and 12.7 ± 2.8 g for *Sparus aurata*. Since both types of fish were cultivated, they are sold with a standard size and weight, which results in a similarity between individuals of the same species. The weight of the gastrointestinal tracts of European sea bass ranged between 8.1 and 16.4 g, while in gilt-head sea breams it ranged between 6.8 and 17.2 g.

### 3.2. Microplastics Occurrence

Table 2 shows the results of the quantification and characterization of extracted MPs from the fish gastrointestinal tracts. As indicated in Section 2, to control airborne contamination, especially that of microfibers, several actions were taken, which included procedural controls within every batch of digested samples. The number of microfibers per control sample analysis was below 3, though in most cases they were orange (from laboratory coats) or absent. On all occasions, the fibres of the same colour were detracted from the ones found in the samples.

**Figure 1:**
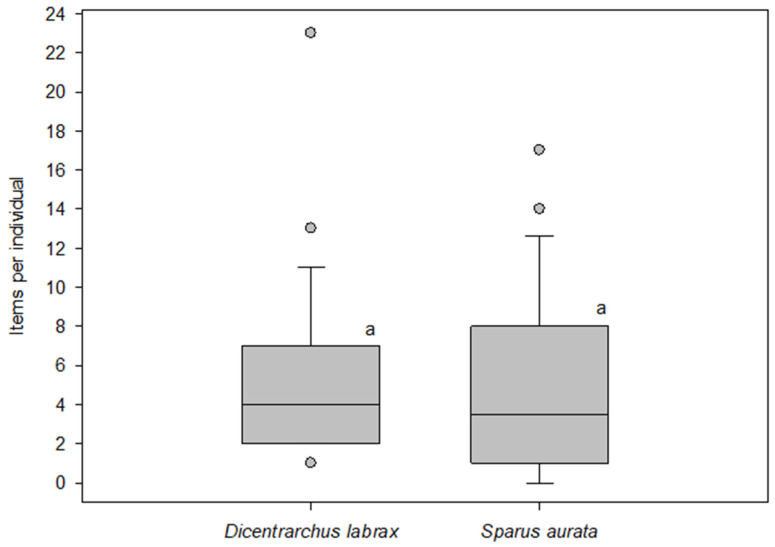
Number of anthropogenic particle items per individual in two aquaculture species of fish, *Dicentrarchus labrax* and *Sparus aurata*; *n* = 42–45; boxes with the same letter indicate no significant differences (*p* > 0.05). The boundary of the box closest to zero indicates the 25th percentile, a line within the box marks the median, and the boundary of the box farthest from zero indicates the 75th percentile. Whiskers (error bars) above and below the box indicate the 90th and 10th percentiles. Circles represent outliers.

A total of 242 particles were found in the 45 samples of *Dicentrarchus labrax*, with the average of items/individual being 5.4 ± 4.2. In the case of *Sparus aurata*, a total of 208 particles were found in the 41 samples, with the average of items/individual being 5.1 ± 5.1 (Figure 1). In the case of *Dicentrarchus labrax,* all the specimens contained MPs except one (2.2%), while in the case of *Sparus aurata* nine analysed specimens did not contain any plastic item (21.9%). Tangled messes were extracted from five *Sparus aurata* individuals, while this material was not observed in specimens of *Dicentrarchus labrax*. Figure 1 shows the box and whiskers plot of the number of items per individual. Statistical analysis revealed that there were not significant differences in particle abundances between both species.

Previous studies have indicated a positive correlation between the concentration of MPs and fish body size [6]. In this case, no significant relationships were observed between the MP content and fish size or intestinal tracts weight probably as a result of the standardized sizes for commercialization purposes.

**Figure 2:**
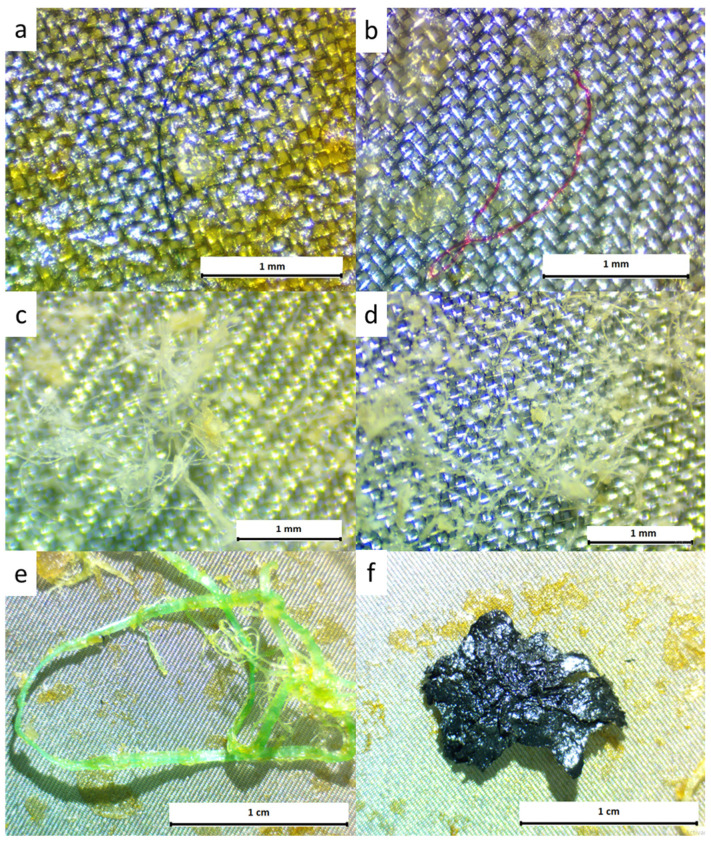
Stereomicroscope photographs of the MPs found in the gastrointestinal tracts of *Dicentrarchus labrax* and *Sparus aurata* analysed in this study; (**a**) blue and (**b**) red microfiber found in the tracts of a *Dicentrarchus labrax* individual; (**c**,**d**) tangled messes found in tracts of *Sparus aurata;* (**e**) line and (**f**) film found in gastrointestinal tract of a *Sparus aurata* individual.

Regarding the MP shapes identified in all the samples, 98.2% were microfibers (*n* = 442), 1.1% tangled messes (*n* = 5), 0.2% lines (*n* = 1), 0.2% films (*n* = 1), and 0.2% fragments (*n* = 1). It should also be indicated that a piece of tar with an average size of 200 µm was extracted from one of the gilt-head sea bream specimens. Figure 2 shows images obtained under the stereomicroscope of different identified MPs forms.

**Figure 3:**
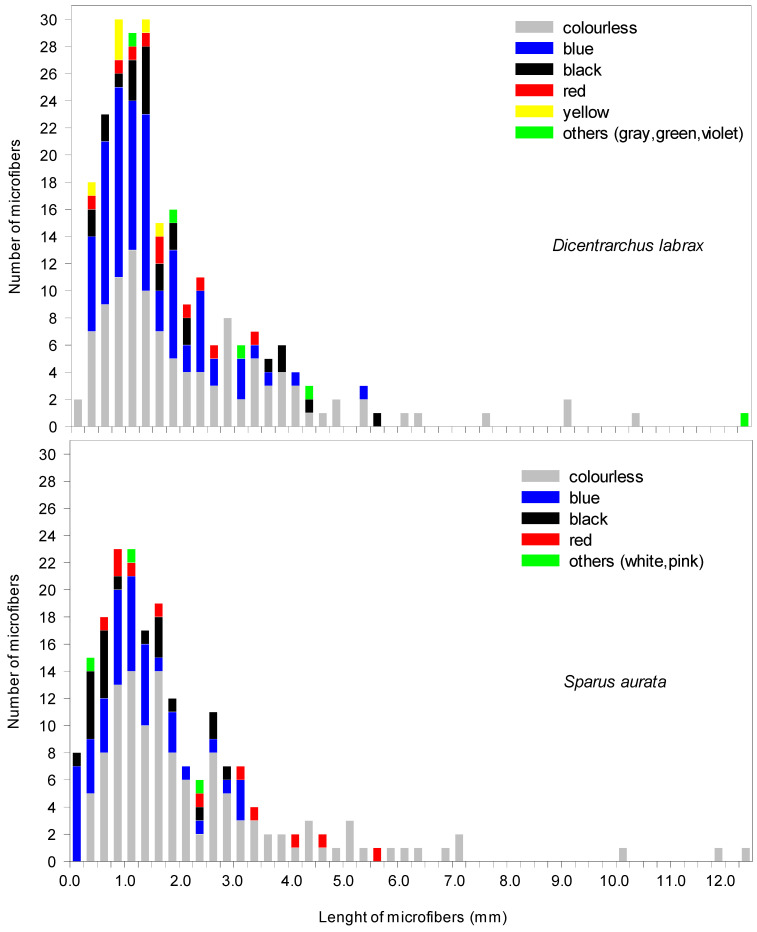
Histogram of size (largest dimension) and colour distribution of the microfibers found in the gastrointestinal tracts of the two species of aquaculture fish evaluated, *Dicentrarchus labrax* and *Sparus aurata*; *n* = 211–242.

Concerning the colour and size of the microfibers, Figure 3 shows the histogram of the distribution of both parameters. As can be seen, colourless microfibers were the most common (47.7% for *Dicentrarchus labrax* and 60.9% for *Sparus aurata)*, followed by blue (35.3% vs. 24.8%, respectively), black (8.7% vs. 7.9%, respectively), and red (4.2% vs. 5.4%, respectively). In the case of *Dicentrarchus labrax*, yellow microfibers (2.5%), grey (0.8%), green (0.4%), and violet (0.4%) were also found, while white (0.5%) and pink (0.5%) microfibers were found in *Sparus aurata*. The size of the microfibers ranged between 50 μm and 12.4 mm, with those of 0.8–1.2 mm being the most abundant. Data show a similar MP shape, colour, and length pattern for both species, which may be related to exposure to the very homogeneous environmental conditions that occur in such fish farms.

### 3.3. Composition of the Microfibers

Sixty-nine microfibers (15.3% of the total number of microfibers found), 28 (40.6%) for European sea bass and 41 (59.5%) for gilt-head sea bream, were randomly selected and analysed by μFTIR spectroscopy as indicated in Section 2. According to the Guidance of Marine Litter in European Seas of the European Commission [20], in MPs studies, formal identification of the polymer composition is not so critical for larger particles (<500 μm) while for smaller sizes a threshold of 10% of the total number of particles found is recommended to be taken as a reference. Though most of the particles had a size larger than 500 μm, we decided to maintain such a threshold (in our case it was slightly higher, 15.3%) in order to provide a general and realistic overview of the composition of the polymers.

**Figure 4:**
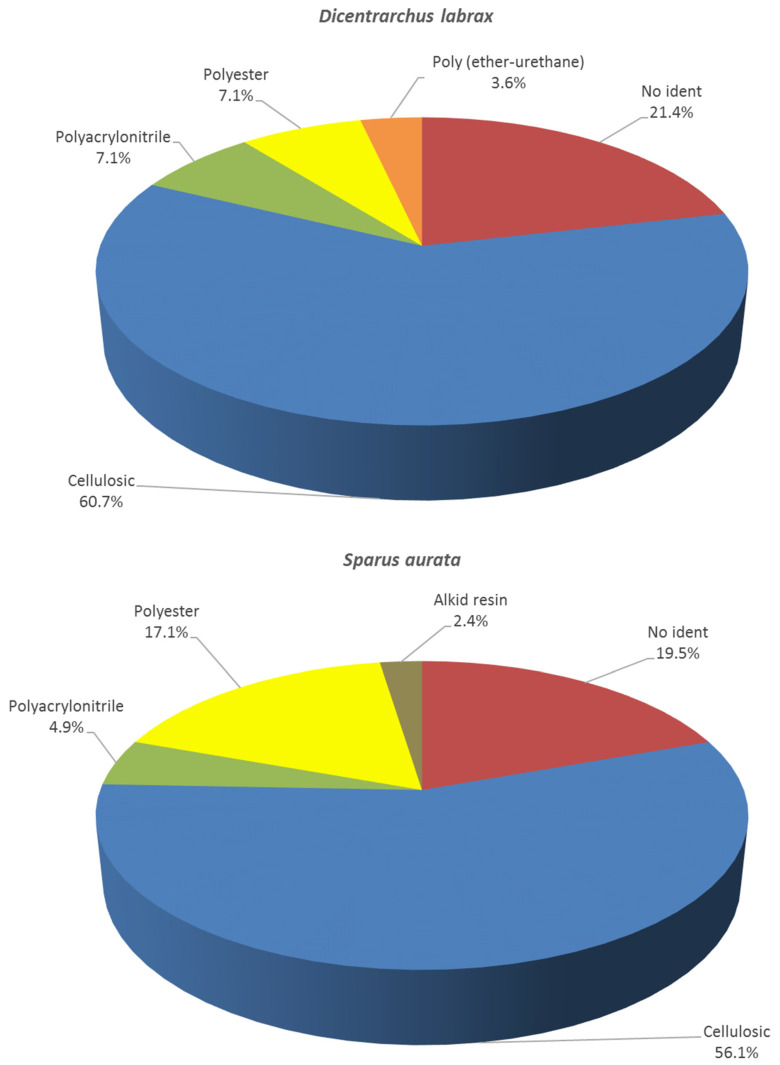
Distribution of the composition of the microfibers found in *Dicentrarchus labrax* (*n* = 41) and *Sparus aurata* within this study (*n* = 45). No-ident: Not identified.

Figure 4 displays the distribution of the composition of the microfibers analysed. Concerning gilt-head sea bream, 23 of them (56.1%) were found to be either natural or semisynthetic cellulose, which is a natural polymer that cannot be formally considered plastic. As indicated in Section 2, as a result of the high similarity of the spectra between natural and semisynthetic cellulose, we have grouped them as cellulosic. Besides, very frequently, commercial libraries mix natural and semi-synthetic celluloses, making their characterization unreliable. In our case, eight of such cellulosic microfibers (34.8%) displayed non-natural colours (red, blue, and black), evidencing some kind of anthropogenic processing. These materials can also be considered of concern as a result of the dyes and other industrial additives that they contain [21]. In the case of European sea bass, 17 of the 28 randomly selected microfibers (60.7%) were also cellulosic, all of them displaying non-natural colours (blue, black and yellow).

Regarding the rest of the microfibers, four polymers accounted for the plastic material: polyester, polyacrylonitrile, alkyd resin, and poly (ether-urethane) (24.4% in the case of gilt-head sea bream and 21.4% of the analysed particles in European sea bass). Some of the particles were not identified (19.5% for gilt-head sea bream and 21.4% for European sea bass) since an acceptable matching percentage was not achieved (see the Experimental Section for more details). Polyester and polyacrylonitrile are widely used in textile fibres for clothing and other industries. Alkyd (which was only found in European sea bass) is also a polyester resin modified by the addition of fatty acids, among others, which are used in commercial coatings (paints, varnishes, etc.). Poly(ether-urethane) has a wide variety of uses, including the fabrication of elastic clothing. Only alkyd resins have a density lower than that of sea water.

### 3.4. Comparison with Previous Studies Published in the Literature

The shape, colour, size, and composition pattern of the MPs found in both types of fishes clearly match with previous studies carried out in the Canary Islands region regarding the determination of MPs in the marine environment. As an example, Villanova-Solano et al. studied the presence of MPs in sublittoral coastal sediments (5–7 depth) of La Palma island [22], finding the prevalence of colourless (86.0%) and blue (9.8%) microfibres (microfibers accounted for 98.3%), most of which were cellulosic (81.3%), although polyester, nylon, and acrylic fibres (polyacrylonitrile) were also found. The average size of the microfibers was 2423 ± 2235 µm. Sevillano-González et al. studied for the first time the presence of MPs in *Diadema Africanum* sea urchins collected at two locations of Tenerife, 32 km from the fish farms where specimens evaluated in the present study were reared [23]. The analysis of 33 individuals showed that 97.5% of the items were microfibers (average size of 1642 ± 1616 μm), mainly blue (43.3 and 47.0% in the two sampling points) and also colourless (32.5 and 39.5%). Regarding the composition, they were mainly cellulosic (46.0%), polypropylene (24.3%), and polyethylene terephthalate (24.3%). Very recently, Pérez-Reverón et al. also determined the presence of MPs in recycled wastewater from the Canary Islands [24], finding in the final effluent of five wastewater treatment plants a high prevalence of cellulosic (44.0%) and polyester (18.0%) microfibers (84.4–100%), mainly colourless (15.0–33.3%) and blue (40.0–55.6%). The average size of the fibres was 786.9 ± 812.1 μm. The results of all these works are clearly coincident, pointing to a possible common origin: wastewater discharge points, which are widely distributed around the islands.

Table 3 compiles the results of published papers in which MPs have been determined in different fish species of the Atlantic Ocean. The number of items per individual vary among studies (40–0.26 items/individual), blue generally being the most common colour, and microfibers the most abundant MP shape. With regards to the only study from the Canary Islands region assessing MP contamination in farmed European sea bass [7], results show MPs colour and shape distributions similar to those found in the present study. However, a higher variety of polymers were identified (cellulosic and nylon were the most abundant) as well as a lower content of items per individual (1.43 ± 1.75 items/individual). Sixty-five percent of the fish sampled contained MPs. Although they were reared in farms at the same zone as in our study, differences may be attributed to the fact that samples were bought in 2016 and 2017, 2–3 years before our study, as well as a possible variation of MPs presence in such zone. A comparison of results of both studies may indicate an increased presence of MPs in the marine environment surrounding fish farms of the Canary Islands during the latest years. However, further comprehensive temporal contrasts are desirable. In the work of Herrera et al. [25], the gastrointestinal tracts of non-farmed Atlantic chub mackerel (*Scomber colias*) were analysed, finding MPs (mainly blue and dark fibres; composition was not determined) in 78.4% of the sampled fish, the average concentration being 2.17 ± 2.04 items/individual. Both studies, together with our findings, clearly suggest a prevalent presence of MPs in fish gastrointestinal tracts of cultivated and wild fish of the region.

## 4. Conclusions

The presence of anthropogenic particles in gastrointestinal tracts of aquaculture European sea bass (*Dicentrarchus labrax*) and gilt-head sea bream (*Sparus aurata)* has been confirmed in specimens reared in Tenerife (Canary Islands, Spain). The analysed samples contained mainly cellulosic, polyester, polyacrylonitrile, and poly (ether-urethane) microfibers, being mostly colourless and blue, and 97.8% of European sea bass and 78.1% of gilt-head sea bream individuals contained such anthropogenic particles. The colour, size, shape, and composition pattern found clearly agree with previous findings in the marine environment of the region, suggesting a possible common origin, among which wastewater discharges are clearly highlighted. Results show the impact of the anthropogenic contamination of microplastics on fish species, even in controlled aquaculture systems. These findings, added to the importance of the aquaculture industry for providing fisheries resources, highlight the potential entry of microplastics into the food chain through the ingestion of fish, encouraging further monitoring of other farmed fish species of the Atlantic region.

## Figures and Tables

**Table 1:** Fork length, standard length, specimen weight and gastrointestinal tract weight of analysed fish individuals of European sea bass and gilt-head sea breams produced by aquaculture in Tenerife (Canary Islands, Spain). Average (mean ± SD), minimum and maximum values of variables are given.

Species (*n*)	Fork Length (cm)	Standard Length (cm)	Specimen Weight (g)	Gastrointestinal Tracts Weight (g)
Europen sea-bass (*Dicentrarchus labrax*) *n* = 45	37.5 ± 1.4 cm Min = 33.8 cm Max = 41.6 cm	33.7 ± 2.0 cm Min = 30.1 cm Max = 40.6 cm	660.5 ± 46.3 g Min = 536.4 g Max = 787.3 g	11.0 ± 1.6 g Min = 8.1 g Max = 16.4 g
Gilt-head sea bream (*Sparus aurata*) *n* = 41	31.4 ± 2.7 cm Min = 26.0 cm Max = 36.5 cm	27.1 ± 2.3 cm Min = 22.5 cm Max = 34.0 cm	609.7 ± 125.8 g Min = 322.2 g Max = 804.6 g	12.7 ± 2.8 g Min = 6.8 g Max = 17.2 g

**Table 2:** Results of the analysis of the gastrointestinal tracts of *Dicentrarchus labrax* and *Sparus aurata* fishes bought in local markets of Tenerife (Canary Islands, Spain) and reared in aquaculture systems in the southwest of the island.

Species (*n*)	Total Number of Particles Found	Average Items/Individual ± SD	Items/Individual Range	Average Items Length ± SD	Items Length Range	Shape Classification
European seabass (*Dicentrarchus labrax*) *n* = 45	242	5.4 ± 4.2	1–23	1957 ± 1699 μm	221 μm–12.4 mm	242 microfibers (100%)
Gilt-head sea bream (*Sparus aurata*) *n* = 41	208 *	5.1 ± 5.1	0–17	1988 ± 1853 μm	69 μm–12.4 mm	200 microfibers (96.1%) 1 line (0.5%) 1 film (0.5%) 1 fragment (0.5%) 5 tangled messes (2.4%)

* Including tangles messes.

**Table 3:** Comparison of the results obtained in this study with previous ones in which the occurrence of MPs has been determined in commercial fish species at the regions of the Canary Islands or nearby areas.

Location	Species	Number of Individuals Analysed	Digestion	Items/Individual	Shape (%)	Fibers Length	Colours (%)	Chemical Composition	Reference
Tenerife, Canary Islands, Atlantic Ocean	*Dicentrarchus labrax* (farmed)	83	10% KOH three times the organic material, room temperature, 2 weeks	1.43 ± 1.75	Fibers (81.0%) Fragments (12.0%) Films (4.0%) Lines (3.0%)	-	Blue (26.3%) Yellow (23.7%) Black (16.9%) Transparent (14.4%) Pink (5.1%) White (2.5%) Red (2.5%) Green (1.7%) Silver (1.7%) Grey (1.7%) Yellowish semitransparent (1.7%) Brown (0.8%) Purple (0.8%)	**Fibers (11)** Cellulose/Cellophane (55.0%) Nylon (27.0%) Rayon (9.0%) Acrylic (9.0%)] **Particles (20)** PE (25.0%) PP (25.0%) PS (5.0%) SAN (5.0%) PA (5.0%) EPDM (5.0%) E/p (5.0%) EVA (5.0%) Polynorbornene (5.0%) Nitrocellulose (5.0%) Epoxy resin (5.0%) Phenolic resin (5.0%)	[7]
Gran Canaria and Lanzarote, Canary Islands, Atlantic Ocean	*Scomber colias* (wild)	120	10% KOH	2.17 ± 2.04	Fibers (74.2%) Fragments (11.9%) Paints (11.5%) Lines (1.5%) Films (0.8%)	-	Blue (55.0%) Dark/Black (23.5%) Red (10.4%) Green (5.0%) Clear/White (4.6%) Yellow/Brown (1.5%)	-	[19]
Eastern Central Atlantic Ocean, Coast of Ghana	*Sardinella maderensis* (wild)	80	20 mL of 10 M KOH at 60 °C for 24 h	32.0 ± 2.7	Pellets (31.0%) Microbeads (29.0%) Burnt plastic films (22.0%) Clear plastic fragments (6.0%) White plastic fragments (3.0%) Green plastic fragment (5.0%) Thread plastics (2.0%) Microfibers (2.0%) Foams (<0.1%)	-	-	-	[26]
	*Sardinella aurita* (wild)	47		26.0 ± 1.6					
	*Dentex angolensis* (wild)	28		40.0 ± 3.8					
Portuguese coast, Atlantic Ocean	*Sardina pilchardus* (wild)	20	10% KOH at room temperature for 2 days	0.26 ± 0.56	Fibers (71.0%) Fragments (24.0%) Films (6.0%)	11.47 ± 19.05 mm	Blue (35.0%) Transparent (29.0%) Green (12.0%) Black (12.0%) Red and Purple (<6.0%)	-	[27]
	*Trachurus trachurus* (wild)	20		0.37 ± 0.60		0.54 ± 0.53 mm			
	*Scomber spp.* (wild)	13		0.38 ± 0.51		1.13 ± 1.09 mm			
Portuguese coast, Atlantic Ocean	*Trachurus trachurus* (wild)	82	10% KOH	2.24 ± 2.05	Fibers (88.0%) Fragments (12.0%)	1090 ± 1011 µm	Blue (39.0%) Black (25.0%) Red (12.0%) Green (12.0%) Transparent (12.0%)	PET (64.0%) PE (27.0%) PP (9.0%)	[28]
	*Scomber colias* (wild)	82		2.46 ± 4.12	Fibers (70.0%) Fragments (30.0%)			PE (47.0%) PET (34.0%) PP (16.0%) PA (3.0%)	
North and Central Moroccan Atlantic coast	*Trachurus trachurus* (wild)	147	-	0.46 ± 1.29	Fibers (58.0%) Fragments (40.0%) Films (2.0%)	-	Blue (33.0%) White (31.0%) Red (16.0%) Black (11.0%) Green (7.0%) Yellow (2.0%)	Acrylic (47.0%) PS (32.0%) Others (21.0%)	[29]
Arona, Tenerife, Canary Islands, Atlantic Ocean	*Dicentrarchus labrax* (farmed)	45	10% KOH 60 °C 24 h	5.4 ± 4.2	Fibers (100%)	221 µm–12.4 mm	Colourless (47.7%) Blue (35.3%) Black (8.7%) Red (4.2%) Yellow (2.5%) Grey (0.8%) Green (0.4%) Violet (0.4%	Cellulosics (60.7%) Polyester (7.1%) Polyacrylonitrile (7.1%) Poly (ether-urethane) (3.6%) Non identified (21.4%)	This study
	*Sparus aurata* (farmed)	41		5.1 ± 5.1	Fibers (96.1%) Line (0.5%) Tangled messes (2.4%) Films (0.5%) Fragments (0.5%)	69 µm–12.4 mm	Colourless (60.9%) Blue (24.8%) Black (7.9%) Red (5.4%) White (0.5%) Pink (0.5%)	Cellulosics (56.1%) Alkid resin (2.4%) Polyester (17.1%) Polyacrylonitrile (4.9%) Non identified (19.5%)	

## Data Availability

The data presented in this study are available on request from the corresponding author.

## References

[1] Ugwu K., Herrera A., Gómez M. (2021). Microplastics in marine biota: A review. Mar. Pollut. Bull..

[2] Bai C.-L., Liu L.-Y., Hu Y.-B., Zeng E.Y., Guo Y. (2022). Microplastics: A review of analytical methods, occurrence and characteristics in food, and potential toxicities to biota. Sci. Total Environ..

[3] Jiang J.-Q. (2018). Ocurrence of microplastics and its pollution in the environment: A review. Sustain. Prod. Consum..

[4] Vighi M., Bayo J., Fernández-Piñas F., Gago J., Gómez M., Hernández-Borges J., Herrera A., Landaburu J., Muniategui-Lorenzo S., Muñoz A.-R. (2021). Micro and Nano-Plastics in the Environment: Research Priorities for the Near Future. Springer Cham..

[5] Li J., Liu H., Chen P. (2018). Microplastics in freshwater systems: A review on occurrence, environmental effects, and methods for microplastics detection. Water Res..

[6] Cheung L.T.O., Lui C.Y., Fok L. (2018). Microplastic Contamination of Wild and Captive Flathead Grey Mullet (*Mugil cephalus*). Int. J. Environ. Res. Public Health.

[7] Reinold S., Herrera A., Saliu F., Hernández-González C., Martínez I., Lasagni M., Gómez M. (2021). Evidence of microplastics ingestion by cultured European sea bass (*Dicentrachus labrax*). Mar. Pollut. Bull..

[8] Garcia A.G., Suárez D.C., Li J., Rotchell J.M. (2020). A comparison of microplastic contamination in freshwater fish from natural and farmed sources. Environ. Sci. Pollut. Res..

[9] Wu F., Wang Y., Leung J.Y.S., Huang W., Zeng J., Tang Y., Chen J., Shi A., Yu X., Xu X. (2019). Accumulation of microplastics in typical commercial aquatic species: A case study at a productive aquaculture site in China. Sci. Total Environ..

[10] Corami F., Rosso B., Sfristo A.A., Gambaro A., Mistri M., Munari C., Barbante C. (2022). Additives, plasticizers, small microplastics (<100 μm), and other microlitter components in the gastrointestinal tract of commercial teleost fish: Method of extraction, purification, quantification, and characterization using Micro-FTIR. Mar. Pollut. Bull..

[11] Froese R., Pauly D. Fish Database. http://www.fishbase.org.

[12] Toledo-Guedes K., Sánchez-Jerez P., González-Lorenzo G., Brito-Hernández A. (2009). Detecting the degree of establishment of a non-indigenous species in coastal ecosystems: Sea bass *Dicentrarchus labrax* escapes from sea cages in Canary Islands (Northeastern Central Atlantic). Hydrobiologia.

[13] Leitão F., Santos M.N., Erzini K., Monteiro C.C. (2008). The effect of predation on artificial reef juvenile demersal fish species. Mar. Biol..

[14] Asociación Empresarial de Acuicultura de España. https://www.apromar.es.

[15] Bayo J., Rojo D., Martínez-Baños P., López-Castellanos J., Olmos S. (2021). Commercial Gilthead Seabream (*Sparus aurata* L.) from the Mar Menor Coastal Lagoon as Hotspots of Microplastic Accumulation in the Digestive System. Int. J. Environ. Res. Public Health.

[16] Barboza L.G.A., Lopes C., Oliveira P., Bessa F., Otero V., Henriques B., Raimundo J., Caetano M., Vale C., Vale C. (2020). Microplastics in wild fish from North East Atlantic Ocean and its potential for causing neurotoxic effects, lipid oxidative damage, and human health risks associated with ingestion exposure. Sci. Total Environ..

[17] Akoueson F., Sheldon L.M., Danopoulos E., Morris S., Hotten J., Chapman E., Li J., Rotchell J.M. (2020). A preliminary analysis of microplastics in edible versus non-edible tissues from seafood samples. Environ. Pollut..

[18] Hidalgo-Ruz V., Gutow L., Thompson R.C., Thiel M. (2012). Microplastics in the Marine Environment: A review of the methods used for identification and quantification. Environ. Sci. Technol..

[19] Barrows A.P.W., Neumann C.A., Pieper C., Berger M.L., Shaw S.D. (2017). Guide to Microplastic Identification, a Comprehensive Method Guide for Microplastics Identification and Quantification in the Laboratory.

[20] Galgani F., Hanke G., Werner S., Oosterbaan L., Nilsson P., Fleet D., Kinsey S., Thompson R.C., van Franeker J., Vlachogianni T. (2013). Guidance on Monitoring of Marine Litter in European Seas.

[21] Edo C., González-Pleiter M., Tamayo-Belda M., Ortega-Ojeda F.E., Leganés F., Fernández-Piñas F., Rosal R. (2020). Microplastics in sediments of artificially recharged lagoons: Case study in a biosphere reserve. Sci. Total Environ..

[22] Villanova-Solano C., Díaz-Peña F.J., Hernández-Sánchez C., González-Sálamo J., González-Pleiter M., Vega-Moreno D., Fernández-Piñas F., Fraile-Nuez E., Machín F., Hernández-Borges J. (2022). Microplastic pollution in sublittoral coastal sediments of a North Atlantic island: The case of La Palma (Canary Islands, Spain). Chemosphere.

[23] Sevillano-González M., González-Sálamo J., Díaz-Peña F., Hernández-Sánchez C., Catalán-Torralbo S., Ródenas-Seguí A., Hernández-Borges J. (2022). Assessment of microplastic content in Diadema africanum sea urchin from Tenerife (Canary Islands, Spain). Mar. Pollut. Bull..

[24] Pérez-Reverón R., González-Sálamo J., Hernández-Sánchez C., González-Pleiter M., Hernández-Borges J., Díaz-Peña F.J. (2022). Recycled wastewater as a potential source of microplastics in irrigated soils from an arid-insular territory (Fuerteventura, Spain). Sci. Total Environ..

[25] Herrera A., Ŝtindlová A., Martínez I., Rapp J., Romero-Kutzner V., Samper M.D., Montoto T., Aguiar-González B., Packard T., Gómez M. (2019). Microplastic ingestion by Atlantic chub mackerel (*Scomber colias*) in the Canary Islands coast. Mar. Pollut. Bull..

[26] Adikaa S.A., Mahua E., Craneb R., Marchantc R., Montfordd J., Folorunshoe R., Gordon C. (2020). Microplastic ingestion by pelagic and demersal fish species from the Eastern Central Atlantic Ocean, off the Coast of Ghana. Mar. Pollut. Bull..

[27] da Silva J.M., Alves L.M.F., Laranjeiro M.I., Bessa F., Silva A.V., Norte A.C., Lemos M.F.L., Ramos J.A., Novais S.C., Ceia F.R. (2022). Accumulation of chemical elements and occurrence of microplastics in small pelagic fish from a neritic environment. Environ. Pollut..

[28] Pequeño J., Antunes J., Dhimmer V., Bessa F., Sobral P. (2021). Microplastics in Marine and Estuarine Species from the Coast of Portugal. Front. Environ. Sci..

[29] Maaghloud H., Houssa R., Bellali F., El Bouqdaoui K., Ouansafi S., Loulad S., Fahde A. (2021). Microplastic ingestion by Atlantic horse mackerel (*Trachurus trachurus*) in the North and central Moroccan Atlantic coast between Larache (35°30′ N) and Boujdour (26°30′ N). Environ. Pollut..

